# Search for light sterile neutrinos with two neutrino beams at MicroBooNE

**DOI:** 10.1038/s41586-025-09757-7

**Published:** 2025-12-03

**Authors:** The MicroBooNE Collaboration, P. Abratenko, D. Andrade Aldana, L. Arellano, J. Asaadi, A. Ashkenazi, S. Balasubramanian, B. Baller, A. Barnard, G. Barr, D. Barrow, J. Barrow, V. Basque, J. Bateman, O. Benevides Rodrigues, S. Berkman, A. Bhat, M. Bhattacharya, M. Bishai, A. Blake, B. Bogart, T. Bolton, M. B. Brunetti, L. Camilleri, D. Caratelli, F. Cavanna, G. Cerati, A. Chappell, Y. Chen, J. M. Conrad, M. Convery, L. Cooper-Troendle, J. I. Crespo-Anadón, R. Cross, M. Del Tutto, S. R. Dennis, P. Detje, R. Diurba, Z. Djurcic, K. Duffy, S. Dytman, B. Eberly, P. Englezos, A. Ereditato, J. J. Evans, C. Fang, B. T. Fleming, W. Foreman, D. Franco, A. P. Furmanski, F. Gao, D. Garcia-Gamez, S. Gardiner, G. Ge, S. Gollapinni, E. Gramellini, P. Green, H. Greenlee, L. Gu, W. Gu, R. Guenette, P. Guzowski, L. Hagaman, M. D. Handley, O. Hen, C. Hilgenberg, G. A. Horton-Smith, A. Hussain, B. Irwin, M. S. Ismail, C. James, X. Ji, J. H. Jo, R. A. Johnson, Y.-J. Jwa, D. Kalra, G. Karagiorgi, W. Ketchum, M. Kirby, T. Kobilarcik, N. Lane, J.-Y. Li, Y. Li, K. Lin, B. R. Littlejohn, L. Liu, W. C. Louis, X. Luo, T. Mahmud, C. Mariani, D. Marsden, J. Marshall, N. Martinez, D. A. Martinez Caicedo, S. Martynenko, A. Mastbaum, I. Mawby, N. McConkey, L. Mellet, J. Mendez, J. Micallef, A. Mogan, T. Mohayai, M. Mooney, A. F. Moor, C. D. Moore, L. Mora Lepin, M. M. Moudgalya, S. Mulleriababu, D. Naples, A. Navrer-Agasson, N. Nayak, M. Nebot-Guinot, C. Nguyen, J. Nowak, N. Oza, O. Palamara, N. Pallat, V. Paolone, A. Papadopoulou, V. Papavassiliou, H. B. Parkinson, S. F. Pate, N. Patel, Z. Pavlovic, E. Piasetzky, K. Pletcher, I. Pophale, X. Qian, J. L. Raaf, V. Radeka, A. Rafique, M. Reggiani-Guzzo, J. Rodriguez Rondon, M. Rosenberg, M. Ross-Lonergan, I. Safa, D. W. Schmitz, A. Schukraft, W. Seligman, M. H. Shaevitz, R. Sharankova, J. Shi, E. L. Snider, M. Soderberg, S. Söldner-Rembold, J. Spitz, M. Stancari, J. St. John, T. Strauss, A. M. Szelc, N. Taniuchi, K. Terao, C. Thorpe, D. Torbunov, D. Totani, M. Toups, A. Trettin, Y.-T. Tsai, J. Tyler, M. A. Uchida, T. Usher, B. Viren, J. Wang, M. Weber, H. Wei, A. J. White, S. Wolbers, T. Wongjirad, K. Wresilo, W. Wu, E. Yandel, T. Yang, L. E. Yates, H. W. Yu, G. P. Zeller, J. Zennamo, C. Zhang

**Affiliations:** 1 Department of Physics & Astronomy, Tufts University, Medford, MA USA; 2 Department of Physics, Illinois Institute of Technology (IIT), Chicago, IL USA; 3 Department of Physics & Astronomy, The University of Manchester, Manchester, UK; 4 Department of Physics, University of Texas, Arlington, TX USA; 5 School of Physics & Astronomy, Tel Aviv University, Tel Aviv, Israel; 6 Fermi National Accelerator Laboratory (FNAL), Batavia, IL USA; 7 Department of Physics, University of Oxford, Oxford, UK; 8 School of Physics & Astronomy, University of Minnesota, Minneapolis, MN USA; 9 Department of Physics, Imperial College London, London, UK; 10 Department of Physics & Astronomy, Michigan State University, East Lansing, MI USA; 11 Department of Physics, University of Chicago, Chicago, IL USA; 12 Brookhaven National Laboratory (BNL), Upton, NY USA; 13 Department of Physics, Lancaster University, Lancaster, UK; 14 Department of Physics, University of Michigan, Ann Arbor, MI USA; 15 Department of Physics, Kansas State University (KSU), Manhattan, KS USA; 16 Department of Physics & Astronomy, The University of Kansas, Lawrence, KS USA; 17 Department of Physics, University of Warwick, Coventry, UK; 18 Department of Physics, Columbia University, New York, NY USA; 19 Department of Physics, University of California, Santa Barbara, CA USA; 20 SLAC National Accelerator Laboratory, Menlo Park, CA USA; 21 Department of Physics, Massachusetts Institute of Technology (MIT), Cambridge, MA USA; 22 Department of Physics & Astronomy, University of Pittsburgh, Pittsburgh, PA USA; 23 Centro de Investigaciones Energéticas, Medioambientales y Tecnológicas (CIEMAT), Madrid, Spain; 24 Department of Physics, University of Cambridge, Cambridge, UK; 25 Department of Physics & Astronomy, Universität Bern, Bern, Switzerland; 26 Argonne National Laboratory (ANL), Lemont, IL USA; 27 Department of Physics, University of Southern Maine, Portland, ME USA; 28 Department of Physics & Astronomy, Rutgers University, Piscataway, NJ USA; 29 Los Alamos National Laboratory (LANL), Los Alamos, NM USA; 30 Departamento de Física Teórica y del Cosmos, Universidad de Granada, Granada, Spain; 31 School of Physics, Nankai University, Tianjin, China; 32 Department of Physics, University of Cincinnati, Cincinnati, OH USA; 33 School of Physics & Astronomy, University of Edinburgh, Edinburgh, United Kingdom; 34 Center for Neutrino Physics, Virginia Tech, Blacksburg, VA USA; 35 Department of Physics, South Dakota School of Mines and Technology (SDSMT), Rapid City, SD USA; 36 Department of Physics & Astronomy, Queen Mary University of London, London, UK; 37 Department of Physics & Astronomy, Louisiana State University, Baton Rouge, LA USA; 38 Department of Physics, Colorado State University, Fort Collins, CO USA; 39 Department of Physics, Indiana University, Bloomington, IN USA; 40 Department of Physics, New Mexico State University (NMSU), Las Cruces, NM USA; 41 Department of Physics, Syracuse University, Syracuse, NY USA

**Keywords:** Experimental particle physics, Theoretical particle physics

## Abstract

The existence of three distinct neutrino flavours, *ν*_e_, *ν*_μ_ and *ν*_τ_, is a central tenet of the Standard Model of particle physics^1,2^. Quantum-mechanical interference can allow a neutrino of one initial flavour to be detected sometime later as a different flavour, a process called neutrino oscillation. Several anomalous observations inconsistent with this three-flavour picture have motivated the hypothesis that an additional neutrino state exists, which does not interact directly with matter, termed as ‘sterile’ neutrino, *ν*_s_ (refs. ^3–9^). This includes anomalous observations from the Liquid Scintillator Neutrino Detector (LSND)^3^ experiment and Mini-Booster Neutrino Experiment (MiniBooNE)^4,5^, consistent with *ν*_μ_ → *ν*_e_ transitions at a distance inconsistent with the three-neutrino picture. Here we use data obtained from the MicroBooNE liquid-argon time projection chamber^10^ in two accelerator neutrino beams to exclude the single light sterile neutrino interpretation of the LSND and MiniBooNE anomalies at the 95% confidence level (CL). Moreover, we rule out a notable portion of the parameter space that could explain the gallium anomaly^6–8^. This is one of the first measurements to use two accelerator neutrino beams to break a degeneracy between *ν*_e_ appearance and disappearance, which would otherwise weaken the sensitivity to the sterile neutrino hypothesis. We find no evidence for either *ν*_μ_ → *ν*_e_ flavour transitions or *ν*_e_ disappearance that would indicate non-standard flavour oscillations. Our results indicate that previous anomalous observations consistent with *ν*_μ_ → *ν*_e_ transitions cannot be explained by introducing a single sterile neutrino state.

## Abstract (web summary)

Data obtained from the MicroBooNE liquid-argon time projection chamber are used to exclude the single light sterile neutrino interpretation of the LSND and MiniBooNE anomalies at the 95% confidence level.

## Main

A broad experimental programme has shown that the three quantum-mechanical eigenstates of neutrino flavour, *ν*_e_, *ν*_μ_ and *ν*_τ_, are related to the three eigenstates of neutrino mass, *ν*_1_, *ν*_2_ and *ν*_3_, by the unitary Pontecorvo–Maki–Nakagawa–Sakata (PMNS) matrix^11,12^. This mixing between flavour and mass states gives rise to the phenomenon of neutrino oscillation, in which neutrinos transition between flavour eigenstates with a characteristic wavelength in $L/{E}_{\nu }\propto {(\Delta {m}_{ji}^{2})}^{-1}$, where *L* is the distance travelled by the neutrino, *E*_*ν*_ is the neutrino energy and $\Delta {m}_{ji}^{2}={m}_{j}^{2}-{m}_{i}^{2}$ is the difference between the squared masses of the mass eigenstates *ν*_*i*_ and *ν*_*j*_. The three known neutrino mass states give rise to two independent mass-squared differences and thus to two characteristic oscillation frequencies that have been well measured with neutrinos from nuclear reactors^13,14^, the Sun^15^, the atmosphere of Earth^16,17^ and particle accelerators^18–20^.

In apparent conflict with the three-neutrino model, several experiments during the past three decades have made observations that can be interpreted as neutrino flavour change with a wavelength much shorter than is possible given only the two measured mass-squared differences^3–9^. These observations are often explained as neutrino oscillations caused by at least one additional mass state, *ν*_4_, corresponding to a mass-squared splitting of $\Delta {m}_{41}^{2}\gtrsim 1{0}^{-2}\,{{\rm{eV}}}^{2}$, which is much greater than the measured $\Delta {m}_{21}^{2}$ and $\Delta {m}_{32}^{2}$. New mass states would require the addition of an equivalent number of new flavour states, in conflict with measurements of the *Z*-boson decay width^21^, which have definitively shown that only three light neutrino flavour states couple to the *Z* boson of the weak interaction. Therefore, these additional neutrino flavour states must be unable to interact through the weak interaction and are thus referred to as ‘sterile’ neutrinos. In this analysis, we focus specifically on light sterile neutrinos—those with masses below at least half the mass of the *Z* boson. It should be noted that the term ‘sterile neutrino’ has also been used to describe new particles, such as heavy right-handed lepton partners, that are potentially more massive than the *Z* boson. However, our study does not directly test these scenarios. The discovery of additional neutrino states would have profound implications across particle physics and cosmology, for example, on our understanding of the origin of neutrino mass, the nature of dark matter and the number of relativistic degrees of freedom in the early universe.

With the addition of a single new mass state *ν*_4_ and a single sterile flavour state *ν*_s_, the PMNS matrix becomes a 4 × 4 unitary matrix described by six real mixing angles *θ*_*ij*_ (1 ≤ *i* < *j* ≤ 4). Oscillations driven by the two measured mass-squared splittings have not had time to evolve for small values of *L*/*E*_*ν*_. The *ν*_μ_ to *ν*_e_ flavour-change probability, ${P}_{{\nu }_{{\rm{\mu }}}\to {\nu }_{{\rm{e}}}}$, and the *ν*_e_ and *ν*_μ_ survival probabilities, ${P}_{{\nu }_{{\rm{e}}}\to {\nu }_{{\rm{e}}}}$ and ${P}_{{\nu }_{{\rm{\mu }}}\to {\nu }_{{\rm{mu}}}}$, can then, to a very good approximation, be described by

1 $${P}_{{\nu }_{{\rm{\mu }}}\to {\nu }_{{\rm{e}}}}={\sin }^{2}(2{\theta }_{{\rm{\mu e}}}){\sin }^{2}\left(\frac{\Delta {m}_{41}^{2}L}{4{E}_{\nu }}\right),$$

2 $${P}_{{\nu }_{{\rm{e}}}\to {\nu }_{{\rm{e}}}}=1-{\sin }^{2}(2{\theta }_{{\rm{ee}}}){\sin }^{2}\left(\frac{\Delta {m}_{41}^{2}L}{4{E}_{\nu }}\right),$$

3 $${P}_{{\nu }_{{\rm{\mu }}}\to {\nu }_{{\rm{\mu }}}}=1-{\sin }^{2}(2{\theta }_{{\rm{\mu \mu }}}){\sin }^{2}\left(\frac{\Delta {m}_{41}^{2}L}{4{E}_{\nu }}\right),$$

where *θ*_ee_ ≡ *θ*_14_, ${\sin }^{2}(2{\theta }_{{\rm{\mu \mu }}})\equiv 4{\cos }^{2}{\theta }_{14}{\sin }^{2}{\theta }_{24}(1-{\cos }^{2}{\theta }_{14}{\sin }^{2}{\theta }_{24})$ and ${\sin }^{2}(2{\theta }_{{\rm{\mu e}}})\equiv {\sin }^{2}(2{\theta }_{14}){\sin }^{2}{\theta }_{24}$, following the common parameterization^22^. Flavour transitions due to these new oscillation parameters are experimentally probed by observing unexpected deficits or excesses in charged current (CC) *ν*_e_ and *ν*_μ_ interactions in a flavour-sensitive neutrino detector from a source of well-defined neutrino flavour content.

Observations compatible with a fourth neutrino mass state have been made in measurements of intense electron-capture decay sources^6–8^, in which a deficit in detected *ν*_e_ rates implies non-unity ${P}_{{\nu }_{{\rm{e}}}\to {\nu }_{{\rm{e}}}}$ from a $\Delta {m}_{41}^{2} > {\mathcal{O}}(1\,{{\rm{eV}}}^{2})$. Although a hint of non-unity ${P}_{{\overline{\nu }}_{{\rm{e}}}\to {\overline{\nu }}_{{\rm{e}}}}$ is provided by the nuclear-reactor-based Neutrino-4 experiment^9^, this result is in conflict with other reactor-based observations from DANSS, NEOS, PROSPECT and STEREO, which see no evidence for *L*/*E*_*ν*_-dependent ${\overline{\nu }}_{{\rm{e}}}$ disappearance^23–26^. Two accelerator-based experiments, LSND and MiniBooNE, have observed potential evidence of non-zero ${P}_{{\nu }_{{\rm{\mu }}}\to {\nu }_{{\rm{e}}}}$ associated with large mass splittings of $\Delta {m}_{41}^{2} > {\mathcal{O}}(1{0}^{-2}\,{{\rm{e}}{\rm{V}}}^{2})$. The LSND experiment observed an anomalous excess of ${\overline{\nu }}_{{\rm{e}}}$ interactions in a π^+^ decay-at-rest beam^3^. The MiniBooNE experiment, situated downstream from the Booster Neutrino Beam (BNB) proton target facility generating a beam of GeV-scale *ν*_μ_ and ${\overline{\nu }}_{{\rm{\mu }}}$ from decays of boosted π^+^ and π^−^, observed an excess of electromagnetic showers indicative of *ν*_e_ interactions that would imply a non-zero ${P}_{{\nu }_{{\rm{\mu }}}\to {\nu }_{{\rm{e}}}}$ (refs. ^4,5^). Observations of *ν*_e_ disappearance and *ν*_e_ appearance should be accompanied by *ν*_μ_ disappearance (non-unity ${P}_{{\nu }_{{\rm{\mu }}}\to {\nu }_{{\rm{\mu }}}}$) if the PMNS matrix is unitary. No conclusive observation of this *ν*_μ_ disappearance has been reported^27–29^. The overall picture of the existence and phenomenology of sterile neutrino states thus remains inconclusive.

In this article, we present new results on sterile neutrino oscillations from the MicroBooNE liquid-argon time projection chamber (LArTPC) experiment at Fermilab^10^. Situated along the same BNB beamline hosting the MiniBooNE experiment, MicroBooNE was conceived to directly test the non-zero ${P}_{{\nu }_{{\rm{\mu }}}\to {\nu }_{{\rm{e}}}}$ observation of MiniBooNE. By supplanting the Cherenkov detection technology of MiniBooNE with the precise imaging and calorimetric capabilities of a LArTPC, MicroBooNE can reduce backgrounds and select a high-purity sample of true *ν*_e_-generated final-state electrons. The first *ν*_e_ measurement results of MicroBooNE using differing final-state topologies showed no evidence for an excess of *ν*_e_-generated electrons from the BNB^30–33^. These results were used to set limits on *ν*_μ_ → *ν*_e_ flavour transitions, excluding sections of the region in $(\Delta {m}_{41}^{2},{\sin }^{2}(2{\theta }_{{\rm{\mu e}}}))$ space favoured by LSND and MiniBooNE data^34^. As the BNB has an intrinsic contamination of electron neutrinos, the disappearance of electron neutrinos can cancel the appearance of electron neutrinos from *ν*_μ_ → *ν*_e_ oscillations^35^. This effect leads to a degeneracy between the impact of the mixing angles *θ*_μe_ and *θ*_ee_ of equations (1) and (2) that weakens the sensitivity to the parameters of the expanded 4 × 4 PMNS matrix.

**Fig. 1:**
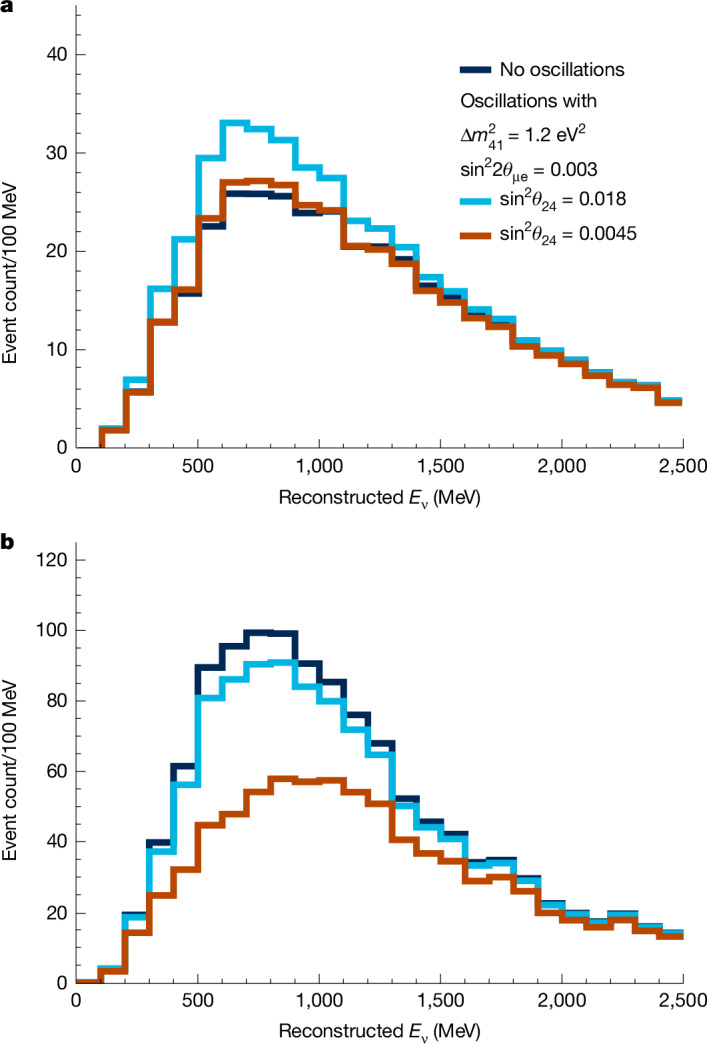
Illustration of the breaking of the degeneracy between *ν*_e_ appearance and disappearance enabled by the independent BNB and NuMI datasets in MicroBooNE. **a**,**b**, Simulated reconstructed energy spectra of FC CC *ν*_e_ interactions in MicroBooNE from the BNB (**a**) and the NuMI beam (**b**). The dark blue histograms show the 3*ν* expectation for ${\sin }^{2}{\theta }_{24}={\sin }^{2}(2{\theta }_{{\rm{\mu e}}})=0$. The light blue and red histograms show expectations for two sets of parameters of the 4*ν* model, both with $\Delta {m}_{41}^{2}=1.2\,{{\rm{e}}{\rm{V}}}^{2}$ and ${\sin }^{2}(2{\theta }_{{\rm{\mu e}}})=0.003$. The light blue histograms show the expectation for ${\sin }^{2}{\theta }_{24}=0.018$ and the red histograms show the expectation for ${\sin }^{2}{\theta }_{24}=0.0045$. Note that these parameters were chosen specifically to highlight differences in the oscillated spectra between BNB and NuMI and do not imply that parameter spaces associated with these values are newly excluded by this result.

We overcome the limitations of the degeneracy between *ν*_e_ appearance and *ν*_e_ disappearance by performing one of the first oscillation searches using two accelerator neutrino beams: the BNB and the Neutrinos at the Main Injector (NuMI) beam. The MicroBooNE detector is aligned with the direction of BNB and is at an angle of about 8° relative to the NuMI beam. Beam timing information is used to distinguish and record events from each beam separately. This configuration results in two neutrino datasets differing in the intrinsic electron-flavour fraction. The electron-flavour content of BNB is 0.57% and that of the NuMI beam is 4.6%. These two independent sets of data, with substantially different electron-flavour contents, break the degeneracy between *ν*_e_ appearance and disappearance. We show the impact of using two beams in Fig. 1a,b, in which we compare simulated *ν*_e_ energy spectra from the BNB and the NuMI beam for the three-flavour (3*ν*) hypothesis and for two sets of parameters of the expanded four-flavour (4*ν*) PMNS model with $\Delta {m}_{41}^{2}=1.2\,{{\rm{e}}{\rm{V}}}^{2}$ and ${\sin }^{2}(2{\theta }_{{\rm{\mu e}}})=0.003$. For ${\sin }^{2}{\theta }_{24}=0.0045$ the *ν*_e_ appearance and disappearance cancel in the BNB, leaving a *ν*_e_ spectrum that is almost identical to the 3*ν* case, whereas the NuMI beam shows an indication of *ν*_e_ disappearance. The appearance and disappearance effects almost fully cancel in the NuMI beam for ${\sin }^{2}{\theta }_{24}=0.018$, whereas the BNB shows a clear indication of *ν*_e_ appearance. In the Methods, we provide further discussion of this degeneracy over a broader range of mass-squared splittings and mixing angles.

Using the two-beam technique, this new MicroBooNE analysis achieves marked improvements in sensitivity to the parameters ${\sin }^{2}(2{\theta }_{{\rm{ee}}})$ and ${\sin }^{2}(2{\theta }_{{\rm{\mu e}}})$ relative to MicroBooNE’s prior sterile neutrino analysis over a broad range of $\Delta {m}_{41}^{2}$ values. These improvements are shown by the sensitivities presented in Extended Data Fig. 2. The results presented here using two neutrino beams place robust new constraints on the validity of the sterile neutrino hypothesis in explaining existing short-baseline anomalies in neutrino physics. This analysis strengthens the direct test of the sterile neutrino interpretation of the MiniBooNE anomaly and allows MicroBooNE to probe the ${\sin }^{2}(2{{\theta }}_{{\rm{\mu }}{\rm{e}}})$ parameter space favoured by LSND. We also constrain ${\sin }^{2}(2{\theta }_{{\rm{ee}}})$, complementing existing exclusions from reactor, solar^36,37^ and β-decay^38^ experiments, thereby further restricting the sterile neutrino parameter space relevant to the gallium anomaly.

We use data corresponding to 6.369 × 10^20^ protons on target (POT) in the BNB, with magnetic van der Meer horns configured to focus positively charged hadrons, leading to a *ν*_μ_-dominated beam with a 5.9% ${\overline{\nu }}_{{\rm{\mu }}}$ component and a 0.57% ${\nu }_{{\rm{e}}}+{\overline{\nu }}_{{\rm{e}}}$ component. From the NuMI beam, a total of 10.54 × 10^20^ POT are used, in which 30.8% were taken with horns configured to focus positively charged hadrons and the remainder with horns focusing negatively charged hadrons. The NuMI flux observed in the MicroBooNE detector, with both horn configurations combined, is *ν*_μ_ dominated with a 42.1% ${\overline{\nu }}_{{\rm{\mu }}}$ component and a 4.6% ${\nu }_{{\rm{e}}}+{\overline{\nu }}_{{\rm{e}}}$ component. In the rest of this paper, we do not discriminate between neutrinos and antineutrinos and refer to the ${\nu }_{{\rm{\mu }}}+{\overline{\nu }}_{{\rm{\mu }}}$ and ${\nu }_{{\rm{e}}}+{\overline{\nu }}_{{\rm{e}}}$ samples as *ν*_μ_ and *ν*_e_ samples for brevity. For both BNB and NuMI, the POT used in this analysis represent roughly half of the total data collected by the MicroBooNE detector; additional data remain available for future studies.

The LArTPC detector of MicroBooNE has an active volume of 10.4 × 2.6 × 2.3 m^3^ containing 85 tonnes of liquid argon. Charged particles passing through the argon create ionization trails. A 273 V cm^−1^ electric field drifts the ionization electrons towards an anode plane consisting of three layers of wires separated by 3 mm and each with a 3-mm wire pitch that collects the electrons and enables three-dimensional imaging of the neutrino interactions. The passage of charged particles through the argon also produces scintillation light that is collected by a system of photomultiplier tubes to provide timing information. Signal processing and calibrations of MicroBooNE data are described in refs. ^39–44^.

Neutrino interactions in the LArTPC are reconstructed with the Wire-Cell analysis framework^45^. The techniques for identifying and reconstructing neutrino interactions and their energies have been described elsewhere^33^. We select a sample of CC *ν*_e_ interactions from the BNB (NuMI beam) with 82% (91%) purity and 46% (42%) efficiency, and a sample of CC *ν*_μ_ interactions with 92% (78%) purity and 68% (62%) efficiency. The CC *ν*_e_ and CC *ν*_μ_ samples are divided into fully contained (FC) and partially contained (PC) samples, depending on whether all charge depositions are contained in a fiducial volume 3 cm within the TPC boundary. The CC *ν*_μ_ events that contain a reconstructed π^0^ are separated into two additional FC and PC samples per beam. Neutral current (NC) interactions that produce a π^0^ are distinguished by the absence of a long muon-like track and the presence of detached reconstructed electromagnetic showers. These form an additional sample. In total, we define 14 distinct event categories, seven for each beam.

We produce a Monte Carlo prediction of our 14 samples, to which we compare the data. There is substantial systematic uncertainty creating this Monte Carlo simulation. The uncertainty on the predicted rates of the 14 samples is given in Table 1 and is referred to as the unconstrained systematic uncertainty. The largest uncertainties come from neutrino interaction modelling for the BNB samples and from a combination of neutrino flux and interaction uncertainties for the NuMI samples. Many of these uncertainties are highly correlated. Thus, a combined fit of all samples effectively constrains the uncertainties on the CC *ν*_e_ prediction and at the same time allows the CC *ν*_e_ prediction to be modified, as can be seen from Table 1. The pionless samples constrain uncertainties on CC *ν*_e_ signal events, whereas the π^0^ samples constrain uncertainties on the dominant background.

**Table 1:** Event counts and systematic uncertainties

	BNB FC	BNB PC	NuMI FC	NuMI PC
Predicted and observed events in CC *ν*_e_ signal samples				
Unconstrained true CC *ν*_e_	310.9	175.7	1107.7	554.2
Constrained true CC *ν*_e_ signal	346.9	188.2	1446.5	703.1
Background	53	40.7	79.2	104.7
Constrained total CC *ν*_e_ samples	399.9	228.9	1525.7	807.8
Data	338	219	1490	824
Systematic uncertainties on CC *ν*_e_ signal samples				
Neutrino flux prediction	5.9%	6.1%	19.6%	19.7%
Neutrino interaction uncertainties	14.7%	14.0%	17.5%	15.1%
Detector uncertainties	3.3%	3.2%	2.0%	3.9%
Monte Carlo statistics	1.57%	1.95%	1.16%	1.67%
Total unconstrained uncertainty	16.3%	15.8%	26.4%	25.2%
Total constrained uncertainty	4.5%	5.5%	5.8%	5.9%

The predicted and observed event counts and the unconstrained and constrained systematic uncertainties on the FC and PC CC *ν*_e_ samples from the BNB and the NuMI beam. All the expected numbers correspond to 6.369 × 10^20^ POT for BNB and 10.54 × 10^20^ POT for NuMI.

Uncertainties on the neutrino flux prediction arise from uncertainties in the production of charged pions and kaons in the BNB and NuMI targets and the material around the target halls and hadron-decay volumes. These uncertainties are evaluated through comparison with external hadron production data^46–48^, following a procedure similar to that described in ref. ^49^. The *ν*_e_ flux from three-body K and μ decays is highly correlated with the *ν*_μ_ flux from two-body π and K decays, allowing our *ν*_μ_ samples to effectively constrain the uncertainties on the *ν*_e_ flux predictions. The neutrino interaction model is tuned using datasets of pionless CC interactions from the T2K experiment^50^. Uncertainties on this neutrino interaction model are evaluated by varying the input parameters within their allowed uncertainties. These uncertainties are correlated between the BNB and NuMI datasets and between the CC *ν*_μ_ and *ν*_e_ samples because of the lepton universality of the weak interaction. Uncertainties on the simulation of the detector include uncertainties on the response of the detector to ionization, uncertainties on the amount of ionization charge freed by passing charged particles through the detector, uncertainties on the electric field map of the TPC, uncertainties on the production and propagation of scintillation light, uncertainties on backgrounds from interactions occurring outside the cryostat, and uncertainties on finite statistics of the simulation samples used for predictions.

**Fig. 2:**
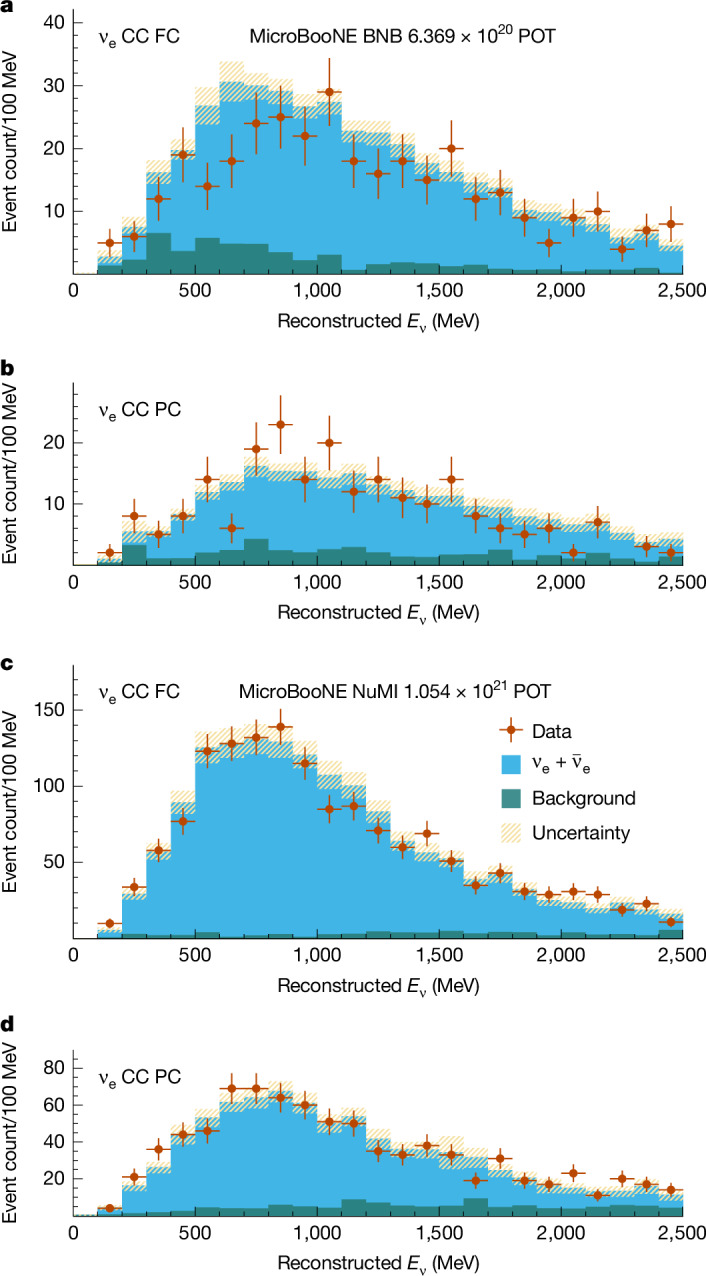
Observed CC *ν*_e_ candidate events. **a**–**d**, Reconstructed energy spectra of events selected as FC CC *ν*_e_ candidates in the BNB (**a**), PC CC *ν*_e_ candidates in the BNB (**b**), FC *ν*_e_ candidates in the NuMI beam (**c**) and PC *ν*_e_ candidates in the NuMI beam (**d**). The data points are shown with statistical error bars. The constrained predictions for each sample are shown for the 3*ν* hypothesis as the solid histograms, with the blue showing the true CC *ν*_e_ events and the green showing the background events. The background category contains CC *ν*_μ_ interactions, NC neutrino interactions, cosmic rays and interactions occurring outside the fiducial volume of the detector. The yellow band shows the total constrained systematic uncertainty on the prediction.

The simultaneous fit to the 14 samples from the BNB and the NuMI beam incorporates all sources of systematic uncertainty through a covariance matrix. We allow ${\sin }^{2}(2{\theta }_{{\rm{\mu e}}})$, ${\sin }^{2}(2{\theta }_{{\rm{ee}}})$ and $\Delta {m}_{41}^{2}$ complete freedom within unitarity bounds as parameters of the fit. The covariance-matrix formalism *χ*^2^ test of the fit can be found in the Methods. The constrained predictions shown in Fig. 2 assume the 3*ν* hypothesis of ${\sin }^{2}(2{\theta }_{{\rm{\mu e}}})={\sin }^{2}(2{\theta }_{{\rm{ee}}})=0$. They agree well with the data, with a *P*-value of 0.92. The best-fit values for the oscillation parameters in the 4*ν* hypothesis are $\Delta {m}_{41}^{2}=1.30\times 1{0}^{-2}\,{{\rm{e}}{\rm{V}}}^{2}$, ${\sin }^{2}(2{\theta }_{{\rm{\mu e}}})=0.999$, and ${\sin }^{2}(2{\theta }_{{\rm{ee}}})=0.999$, with a *χ*^2^ difference with respect to the 3*ν* hypothesis of

4 $$\Delta {\chi }^{2}={\chi }_{{\rm{null}},3\nu }^{2}-{\chi }_{\min ,4\nu }^{2}=0.228.$$

We observe no marked preference for the existence of a sterile neutrino with a *P*-value of 0.96 evaluated using the Feldman–Cousins procedure.

**Fig. 3:**
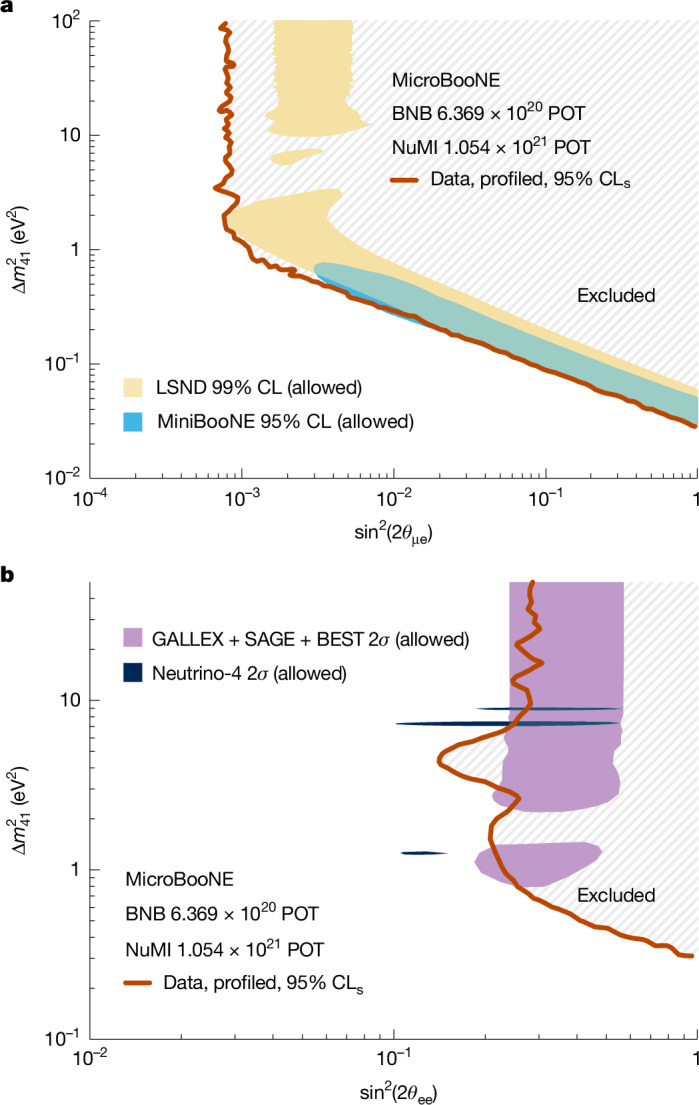
Constraints on parameters of the 4*ν* oscillation model. **a**,**b**, The red lines show exclusion limits at the 95% CL_s_ level in the plane of $\Delta {m}_{41}^{2}$ and ${\sin }^{2}(2{\theta }_{{\rm{\mu }}{\rm{e}}})$ (**a**) or ${\sin }^{2}(2{\theta }_{{\rm{e}}{\rm{e}}})$ (**b**). All the regions to the right of these lines are excluded by the MicroBooNE data. In **a**, the yellow shaded area is the LSND 99% CL allowed regions^3^, which neglects the degeneracy between *ν*_e_ disappearance and appearance. The light blue area is the MiniBooNE 95% CL allowed region^58^, considering both *ν*_e_ disappearance and appearance. In **b**, the purple shaded area is the 2*σ* allowed region of the gallium anomaly^59^. The dark blue shaded area is the 2*σ* allowed region from the Neutrino-4 experiment^9^. For context, note that the stronger-than-expected constraint on ${\sin }^{2}(2{\theta }_{{\rm{\mu e}}})$, driven by the deficit observed in the BNB *ν*_e_ CC FC sample and the excess in the NuMI *ν*_μ_ CC sample, is discussed in detail in the Methods and Extended Data Fig. 2.

Exclusion contours are calculated using the frequentist CL_s_ (confidence level as a function of s) method^51^. The exclusion contour in any two-dimensional parameter space is obtained by profiling the third free parameter. At any point in the two-dimensional space, the value of the profiled parameter that minimizes the *χ*^2^ with respect to the data is chosen. Figure 3a shows the 95% CL_s_ exclusion contour in the $(\Delta {m}_{41}^{2},{\sin }^{2}(2{\theta }_{{\rm{\mu e}}}))$ parameter space. The region allowed at 99% CL by the LSND measurement and the vast majority of the region allowed at the 95% CL by the MiniBooNE experiment are excluded. Figure 3b shows the 95% CL_s_ exclusion contour in the $(\Delta {m}_{41}^{2},{\sin }^{2}(2{\theta }_{{\rm{ee}}}))$ parameter space. A notable portion of the region allowed by gallium measurements and part of the region derived from the Neutrino-4 measurement are excluded. In the Methods and Extended Data Fig. 2, we compare our exclusions with the expected median sensitivities.

In summary, using data from the MicroBooNE detector, we report one of the first searches for a sterile neutrino using two accelerator neutrino beams. The oscillation fit to the 4*ν* model using a total of 14 CC *ν*_e_, CC *ν*_μ_ and NC π^0^ samples from the BNB and the NuMI beam in a single detector achieves a marked reduction of systematic uncertainties and a powerful mitigation of degeneracies between *ν*_e_ appearance and disappearance. The result shows no evidence of oscillations induced by a single sterile neutrino and is consistent with the 3*ν* hypothesis with a *P*-value of 0.96. We comprehensively exclude at a 95% CL the 4*ν* parameter space that would explain the LSND and MiniBooNE anomalies through the existence of a light sterile neutrino in a model with an extended 4 × 4 PMNS matrix. Our result expands the diverse range of experimental approaches, excluding regions that would explain the gallium anomaly and the Neutrino-4 observation with a light sterile neutrino. This work, therefore, provides a robust exclusion of a single light sterile neutrino as an explanation for the array of short-baseline neutrino anomalies observed over the past three decades, representing the strongest constraint from a short-baseline experiment using accelerator-produced neutrinos. Expanded models, including several light sterile neutrinos^52^, neutrino decay effects^53,54^ or production and decay of new particles connected with the dark sector^55,56^ might explain the anomalies. The Short Baseline Neutrino (SBN) Programme^57^ at Fermilab adds two new LArTPC detectors in the BNB, at different distances from the proton target. Future measurements by MicroBooNE and the broader SBN Programme can shed light on this expanded model space, with future comprehensive insights provided by near-term short-baseline measurements from diverse flavour channels and energy regimes.

## Methods

### Neutrino beams at MicroBooNE

The BNB and the NuMI are conventional neutrino beamlines that use intense proton beam pulses to generate on-axis neutrino fluxes peaking in the neutrino-energy range 0.5–5 GeV. Boosted charged mesons escaping the target are focused into a decay pipe using magnetic horns, allowing decaying mesons to impart much of their kinetic energy directly to their neutrino product. The MicroBooNE detector is on the axis of the BNB, 468.5 m from the proton target. The detector is about 8° off-axis and 679 m from the proton target of the NuMI beam. The muon flavour components of these beams are mostly generated via the primary decay channel of the dominant *π* mesons, ${\pi }^{\pm }\to {\mu }^{\pm }+{\nu }_{{\rm{\mu }}}({\overline{\nu }}_{{\rm{\mu }}})$, whereas the electron-flavour component is generated by decay of the pion’s boosted *μ*^±^ daughter, by ${\mu }^{\pm }\to {e}^{\pm }+{\overline{\nu }}_{{\rm{\mu }}}({\nu }_{{\rm{\mu }}})+{\nu }_{{\rm{e}}}({\overline{\nu }}_{{\rm{e}}})$, and by semileptonic decays of sub-dominant K mesons, specifically ${K}^{\pm }\to {\pi }^{0}+{e}^{\pm }+{\nu }_{{\rm{e}}}({\overline{\nu }}_{{\rm{e}}})$ and ${K}_{{\rm{L}}}^{{\rm{0}}}\to {\pi }^{\mp }+{e}^{\pm }+{\nu }_{{\rm{e}}}({\overline{\nu }}_{{\rm{e}}})$.

Neutrinos in the BNB are created by colliding protons with a kinetic energy of 8 GeV on a beryllium target, whereas in the NuMI beamline, 120 GeV protons collide with a carbon target. These differences serve to generate a higher on-axis beam energy in the NuMI beam, as well as a greater proportion of K meson production, leading to a higher *ν*_e_ content at the highly off-axis position of MicroBooNE. The NuMI beam also incorporates a longer charged-meson decay pipe (675 m) than the BNB (50 m), which increases its electron-flavour content by facilitating a higher proportion of decays of secondary μ^+^ from upstream π^+^ decay. Although the latter effect drives higher electron-flavour content on-axis for NuMI relative to the BNB, it is the larger proportion of unfocused or poorly focused K mesons of NuMI that drives its elevated electron-flavour content at the off-axis angle of MicroBooNE relative to the on-axis flux of BNB.

### Neutrino flux simulation

The simulation of the neutrino flux at MicroBooNE accounts for the production of hadrons from the initial interaction of the proton beam on the target and the propagation of these hadrons through a detailed beamline geometry description, achieved using the Geant4 toolkit^60^. Hadron production cross-sections are constrained by dedicated external measurements where available, tailored to the specific beam parameters, including target differences and initial proton beam energy. The BNB simulation, identical to that used at MiniBooNE^61^, uses Geant v.4.10.4 with a custom physics list and constrains π^±^ yields with data from the HARP experiment^62^, along with an updated K^+^ production constraint from SciBooNE^63,64^. The NuMI simulation has been updated to Geant v.4.10.4 with the FTFP-BERT physics list^65^. The constraints on π^±^ and K^±^ yields from the NA49 experiment at CERN^46,47^ are implemented using the PPFX toolkit^49^, which has been updated to use the new Geant version. Uncertainties are estimated for each process and various components of the beamline geometry, resulting in a combined systematic uncertainty of approximately 13% for the BNB and 26% for the NuMI beam on the integrated flux. These uncertainties on the fluxes are different from the uncertainties quoted in Table 1, which are on the overall event rates. These uncertainties are dominated by hadron production rates and are larger for the NuMI beam because of the lack of constraints at large off-axis angles from dedicated hadron production experiments. As the nature of neutrino production in the two beams is different, the uncertainties are considered uncorrelated across the respective beams.

### Degeneracy between *ν*_e_ appearance and disappearance

**Extended Data Fig. 1:**
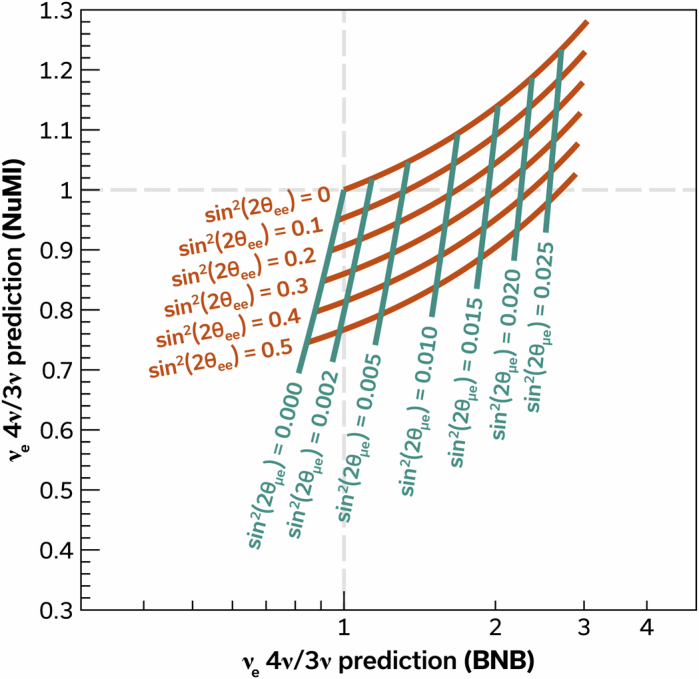
*ν*_e_ event rates in MicroBooNE as a function of oscillation parameters. The *x*-axis (in log scale) shows the expected CC *ν*_e_ event rate in the MicroBooNE detector in the 4*ν* scenario, with respect to the 3*ν* scenario, from the BNB. The *y*-axis (in linear scale) shows the same ratio from the NuMI beam. The lines indicate how these ratios depend on the oscillation parameters of the expanded 4*ν* PMNS matrix for $\Delta {m}_{41}^{2}=1.4\,{{\rm{e}}{\rm{V}}}^{2}$.

The interplay between oscillation of intrinsic *ν*_μ_ and *ν*_e_ components in the BNB and the NuMI beam is shown in Extended Data Fig. 1. For various combinations of the expanded 4*ν* PMNS mixing angles, and assuming $\Delta {m}_{41}^{2}=1.4\,{{\rm{e}}{\rm{V}}}^{2}$, the ratio of predicted *ν*_e_ signal events with 0 < *E*_*ν*_ < 2.5 GeV in MicroBooNE relative to the 3*ν* prediction is shown for the BNB on the *x-*axis and for the NuMI beam on the *y*-axis. By tracing vertically along *x* = 1, we observe that a MicroBooNE BNB *ν*_e_ measurement could be consistent with the 3*ν* hypothesis of *θ*_ee_ = *θ*_μe_ = 0 as well as with non-zero mixing angles in the alternate 4*ν* case. The addition of a NuMI *ν*_e_ measurement enables a much clearer interpretation of the allowed oscillation behaviour, while also strengthening the constraining power of the analysis. Specifically, perfect agreement between data and the 3*ν* prediction for both BNB and NuMI would favour the null oscillation case, whereas a large deficit in the high *ν*_e_-content NuMI beam would indicate competing appearance and disappearance effects in the BNB *ν*_e_ sample. Moreover, we can see that the range of allowed 4*ν* predictions in NuMI and BNB when taken together is quite restricted, allowing us to set tighter limits on this oscillation model based on the observed CC *ν*_e_ event rates after constraints in Table 1 (*x* ≈ 0.9, *y* ≈ 1).

**Extended Data Fig. 2:**
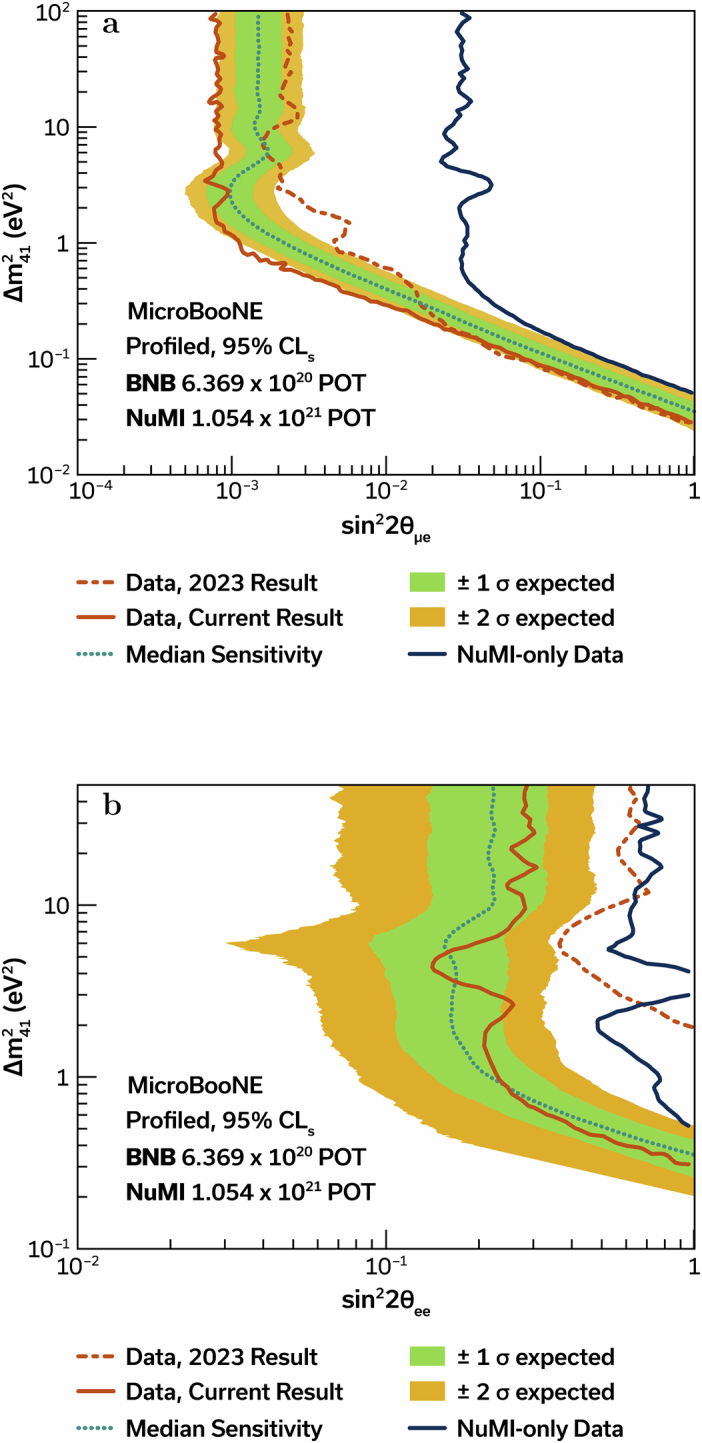
Comparison of the measured exclusions to the expected sensitivities. The red solid lines show the measured exclusion lines at the 95% CL_s_ level in the plane of $\Delta {m}_{41}^{2}$ and (**a**) ${\sin }^{2}(2{\theta }_{{\rm{\mu }}{\rm{e}}})$ or (**b**) ${\sin }^{2}(2{\theta }_{{\rm{e}}{\rm{e}}})$. The blue dashed lines show the median expected sensitivities. The green and yellow bands show the 1*σ* and 2*σ* expected fluctuations around the median sensitivities at each $\Delta {m}_{41}^{2}$ value. The red dashed lines show the previous MicroBooNE BNB-only result^34^ and the blue solid lines shows the measured NuMI-only exclusions at 95% CL_s_.

Extended Data Fig. 2 shows the impact of the degeneracy breaking on the sensitivity to the 4*ν* parameter space. The dashed lines show the exclusion regions of the previous BNB-only analysis of MicroBooNE^34^, compared with the solid red lines, which show the exclusions obtained by this analysis when including the NuMI beam data.

### Statistical methods for oscillation analysis

The combined statistical and systematic uncertainties on the 14 event samples are described by the covariance matrix

5 $$\Sigma =\left(\begin{array}{ccc}{C}_{1,1} & \cdots \, & {C}_{1,14}\\ \vdots  & \ddots  & \vdots \\ {C}_{14,1} & \cdots \, & {C}_{14,14}\end{array}\right),$$

where the *C*_*i*,*j*_ are the bin-by-bin covariance matrices between the *i*th and *j*th event samples. These covariance matrices are the sums of the covariance matrices arising from statistical uncertainties and from each source of systematic uncertainty,

6 $${C}_{i,j}={C}_{i,j}^{{\rm{stat.}}}+\sum _{k}{C}_{i,j}^{{{\rm{syst.}}}_{k}},$$

where the sum runs over the *k* sources of systematic uncertainty. The covariance matrix for the statistical uncertainty follows the Pearson format.

Figure 2 and Table 1 demonstrate the power of the 14 event samples to effectively constrain the systematic uncertainties due to the correlations present in the covariance matrix. To produce the constrained predictions shown in Fig. 2 and the constrained systematic uncertainties in Table 1, a conditional constraint formalism^66^ is used, which uses the 14 event samples simultaneously to constrain the systematic uncertainties and to provide updated predictions for each event sample. To understand how the constraint is applied to the *i*th event sample, the full unconstrained covariance matrix can be written as

7 $$\Sigma =\left(\begin{array}{cc}{C}_{i,i} & {C}_{i,x}\\ {C}_{x,i} & {C}_{x,x}\end{array}\right),$$

where elements with a subscript *x* represent the remaining 13 blocks of the full matrix. An updated, constrained covariance matrix for the *i*th event sample is obtained as

8 $${C}_{i,i}^{{\rm{constr}}.}={C}_{i,i}-{C}_{i,x}\cdot {({C}_{x,x})}^{-1}\cdot {C}_{x,i}.$$

Given the unconstrained binned prediction for the *i*th event sample, *μ*_*i*_, and the remaining binned prediction and data samples, *μ*_*x*_ and *n*_*x*_, a constrained binned prediction can also be formed as

9 $${\mu }_{i}^{{\rm{constr}}.}={\mu }_{i}+{C}_{i,x}\cdot {({C}_{x,x})}^{-1}\cdot ({n}_{x}-{\mu }_{x}).$$

To perform the oscillation fit, a *χ*^2^ test statistic,

10 $${\chi }^{2}={(N-M)}^{{\rm{T}}}\cdot {\Sigma }^{-1}\cdot (N-M),$$

is formed using the 14 binned event samples from the data, *N* = (*n*_1_, …, *n*_14_), where *n*_*i*_ is the *i*th event sample from the data, and the corresponding unconstrained binned predictions for the 14 event samples, *M* = (*μ*_1_, …, *μ*_14_). The oscillation parameters used to produce the prediction are varied until *χ*^2^ is minimized. As the bin-to-bin correlations between the 14 event samples are contained in the matrix Σ, minimizing this *χ*^2^ intrinsically incorporates the constraint procedure into the measurement of the oscillation parameters. As *χ*^2^ is minimized, the absolute systematic uncertainty varies as the number of oscillated neutrino interactions within the fiducial volume changes, whereas the fractional systematic uncertainty remains constant. By contrast, the absolute systematic uncertainty related to non-neutrino backgrounds and out-of-fiducial-volume neutrino interactions remains unchanged. Consequently, the total covariance matrix is updated in accordance with the oscillation parameters of interest.

Exclusion limits on the oscillation parameters are calculated using the frequentist-motivated CL_s_ method^51^, which is commonly used for determining exclusion limits in high-energy physics. The CL_s_ test statistic is defined as CL_s_ = *P*_4*ν*_/*P*_3*ν*_, where *P*_4*ν*_ and *P*_3*ν*_ are the one-sided *P*-values of $\Delta {\chi }_{{\rm{C}}{{\rm{L}}}_{{\rm{s}}},{\rm{d}}{\rm{a}}{\rm{t}}{\rm{a}}}^{2}$ under the 4*ν* and the null 3*ν* hypotheses, respectively, where

$$\Delta {\chi }_{{{\rm{CL}}}_{{\rm{s}}}}^{2}={\chi }_{4\nu }^{2}-{\chi }_{3\nu }^{2}$$

at a given point in the 4*ν* parameter space. These *P*-values are one-sided because the test statistic measures the deviation from the null in the specific direction of the alternative hypothesis. The *P*-values are determined using a frequentist approach by generating pseudo-experiments with the full covariance matrix, assuming the respective hypothesis is true. The region in which CL_*s*_ ≤ 1 − *α* is excluded at the confidence level *α*. By generating pseudo-experiments under the null 3*ν* hypothesis, expected exclusion limits are calculated across the 2D parameter spaces of $\Delta {m}_{41}^{2}$ and ${\sin }^{2}(2{\theta }_{{\rm{\mu e}}})$ or ${\sin }^{2}(2{\theta }_{{\rm{ee}}})$.

To quantify the expected sensitivity of the analysis, the median ${\sin }^{2}(2{\theta }_{{\rm{\mu e(ee)}}})$ value from all expected exclusion limits is determined for each $\Delta {m}_{41}^{2}$ value. These median sensitivities are shown in Extended Data Fig. 2 and are compared with the exclusions set using the data. To show the expected level of fluctuations of the measured limit from the median sensitivity, 1*σ* and 2*σ* bands are also shown in Extended Data Fig. 2, which encompass the central 68.3% and 95.5% of exclusions from the pseudo-experiments. In ${\sin }^{2}(2{\theta }_{{\rm{\mu e}}})$ space, our exclusion is stronger than our median expected sensitivity. Two factors contribute to this stronger exclusion. First, a deficit in the BNB CC *ν*_e_ sample more strongly disfavours *ν*_e_ appearance. Second, the excess in the NuMI CC *ν*_μ_ sample leads to a reduction in the constrained fractional uncertainty on the NuMI *ν*_e_ prediction through the joint fit procedure, which in turn further strengthens the exclusion limit. In ${\sin }^{2}(2{\theta }_{{\rm{ee}}})$ space, the deficit in the BNB CC *ν*_e_ FC sample plays the opposite role, slightly favouring *ν*_e_ disappearance and making the exclusion contour weaker than the median sensitivity.

### Impact of the NuMI CC *ν*_μ_ sideband

**Extended Data Fig. 3:**
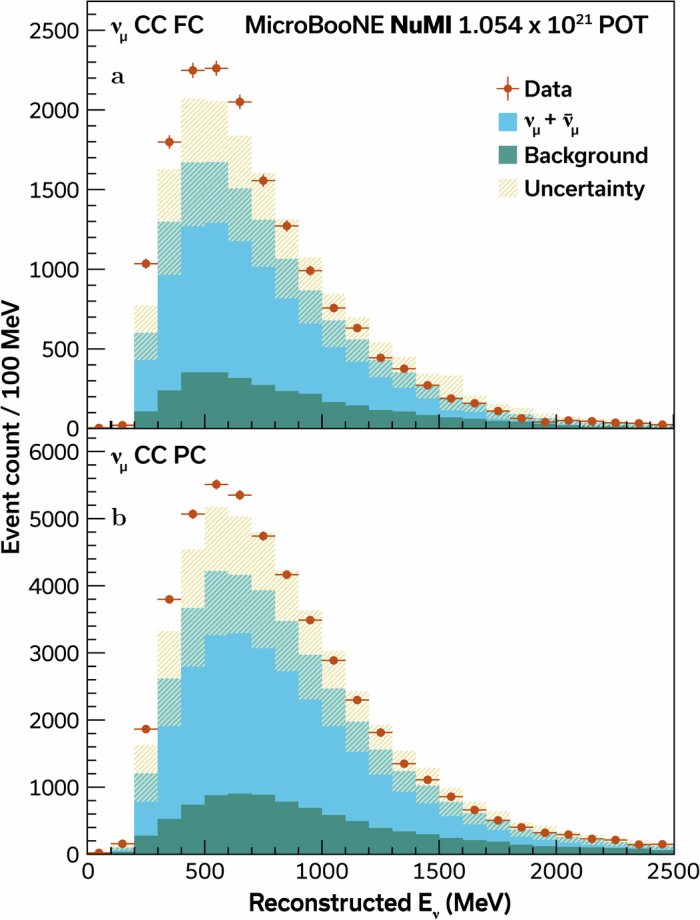
Observed NuMI CC *ν*_μ_ candidate events. Reconstructed energy spectra of events selected as (**a**) fully contained CC *ν*_μ_ candidates in the NuMI beam, and (**b**) partially contained CC *ν*_μ_ candidates in the NuMI beam. The data points are shown with statistical error bars. The constrained predictions for each sample are shown for the 3*ν* hypothesis as the solid histograms, with the blue showing the true CC *ν*_μ_ events and the green showing the background events. The background category contains NC neutrino interactions, cosmic rays, CC *ν*_e_ interactions, and interactions occurring outside the fiducial volume of the detector. The yellow band shows the total systematic uncertainty on the prediction.

As shown in Table 1, a combined fit of the 14 reconstructed samples constrains the signal CC *ν*_e_ prediction and its uncertainties due to the correlations between the sideband and the signal channels. For the NuMI CC *ν*_e_ signal sample in particular, a crucial driver of the constraint is the corresponding CC *ν*_μ_ sideband, shown in Extended Data Fig. 3. Before the fit, the normalization difference between data and the prediction is 24.5% with an overall uncertainty of 21.1% on the prediction.

To evaluate the impact of this difference on the combined fit, we can extend the covariance matrix used in the analysis (364 × 364, corresponding to the energy spectra of the 14 channels) by adding a bin representing the overall NuMI CC *ν*_μ_ normalization (combining FC and PC) and computing the respective covariances with the other 364 analysis bins, resulting in a 365 × 365 matrix. We can then obtain a post-fit mean and error on this normalization parameter by constraining the 364 × 364 block to the data using equations (8) and (9). This gives us an estimate of how much this parameter is effectively being pulled in the combined fit. The post-fit value of this parameter is 1.28 ± 0.058, indicating consistency with the corresponding observed value as well as a modest pull of  about 1.3*σ*.

## Online content

Any methods, additional references, Nature Portfolio reporting summaries, source data, extended data, supplementary information, acknowledgements, peer review information; details of author contributions and competing interests; and statements of data and code availability are available at 10.1038/s41586-025-09757-7.

## Data Availability

The measured data, predicted signal and background, along with their complete systematic uncertainties for the corresponding reconstructed neutrino energy bins in the *ν*_e_ channels, are publicly accessible on HEPData (10.17182/hepdata.166435.v1) and Zenodo (10.5281/zenodo.17161263). Moreover, Δ*χ*^2^ values for each 4*ν* hypothesis across the three-dimensional grid of oscillation parameters are provided.

## Code availability

The MicroBooNE Collaboration is responsible for developing and maintaining the code used for simulating and analysing the raw data that support this result. This code is accessible to the Collaboration members but is not publicly available. Questions about the algorithms and methods used in this analysis can be directed to the corresponding author.
